# Retroperitoneal alveolar rhabdomyosarcoma intruding into spinal canal: A case report and literature review

**DOI:** 10.3389/fmed.2022.1019964

**Published:** 2022-11-03

**Authors:** Yongbai Zhang, Wenpeng Huang, Liming Li, Yongkang Qiu, Hao Jiao, Zhao Chen, Qi Yang, Lele Song, Lei Kang

**Affiliations:** ^1^Department of Nuclear Medicine, Peking University First Hospital, Beijing, China; ^2^Department of Radiology, The First Affiliated Hospital of Zhengzhou University, Zhengzhou, China

**Keywords:** alveolar rhabdomyosarcoma, ^18^F-FDG, PET/CT, retroperitoneum, spinal canal intrusion, case report

## Abstract

**Background:**

Rhabdomyosarcoma (RMS) is the most frequent soft sarcoma in children and adolescents. Alveolar rhabdomyosarcoma (ARMS) is a relatively rare subtype that is characterized by aggressive behavior and an unsatisfactory prognosis. An ARMS can arise anywhere but most commonly occurs at extremity sites with a very small fraction in the retroperitoneum. The utility of 2-Deoxy-2-[fluorine-18]-fluoro-D-glucose (^18^F-FDG) positron emission tomography combined with computed tomography (PET/CT) remains to be established in ARMS.

**Case Report:**

A 3-year-old female child was accidentally found with a large left upper abdominal mass for a day. CT examination indicated a huge soft tissue mass in the left retroperitoneum extending superiorly to the level of the left hilus renalis and inferiorly to the left acetabulum in the pelvic cavity, with intrusion into the lumbar foramens. ^18^F-FDG PET/CT found a mass in the left retroperitoneum from the level of T12 to the left acetabulum, with the maximum standardized uptake value (SUV_max_) of about 7.0, and a CT value of about 39 HU, invading the left L3-5 intervertebral foramina and protruding into the spinal canal, with unclear boundary with the spinal cord. Retroperitoneal tumor resection and the repair operation of vascular exploration were performed. An ARMS was confirmed by postoperative biopsy, immunohistochemical staining, and genetic detection with the rupture of the fork head in rhabdomyosarcoma (FKHR). The patient received chemotherapy and was in a good condition with no recurrence and obvious complications.

**Conclusion:**

Retroperitoneal ARMS is rare and indicates a poor outcome with the potential to involve vital organs and intrude into the spinal canal. Accurate diagnosis and staging using PET/CT would contribute to better risk stratifications and appropriate treatment individually.

## Introduction

Rhabdomyosarcoma (RMS), which is derived from primary mesenchymal cells that differentiate into skeletal muscle, is a type of high-grade rare malignant tumor with an incidence of 4.6 cases per million (1, 2). RMS is usually classified into four histologic subtypes: embryonal rhabdomyosarcoma (ERMS), alveolar rhabdomyosarcoma (ARMS), pleomorphic rhabdomyosarcoma, and sclerosing/spindle cell rhabdomyosarcoma (3). An ARMS is a relatively rare subcategory with a prevalence of about 20–25% of all RMS, compared with ERMS of 50–60% (4). It is more aggressive and has a more unsatisfactory prognosis than other subtypes (5). The ARMS most commonly occurs at extremity sites and sometimes in the head and/or neck or torso, while ERMS typically occurs in the head and neck (6). However, retroperitoneal ARMS is extremely rare and has poor outcomes accompanied by the large tumor volume and frequent involvement of vital organs, leading to difficult resection when it is diagnosed (7). Due to the specific anatomical location, retroperitoneal, especially paraspinal, ARMS shows the potential to infiltrate the vertebrae and protrude into the spinal canal (8, 9).

2-Deoxy-2-[fluorine-18]-fluoro-D-glucose (^18^F-FDG) positron emission tomography combined with computed tomography (PET/CT) is an imaging technology illustrating detailed metabolic and functional molecular information and the precise anatomical region of the lesion (10). It is used in evaluating different tumors and represents an extremely promising investigation method for the diagnosis, staging, and prognosis of rhabdomyosarcoma. Herein, we described a rare case with retroperitoneal ARMS invading the intervertebral foramen and intruding into the spinal canal, and we reviewed the available literature on retroperitoneal ARMS.

## Case presentation

**Figure 1:**
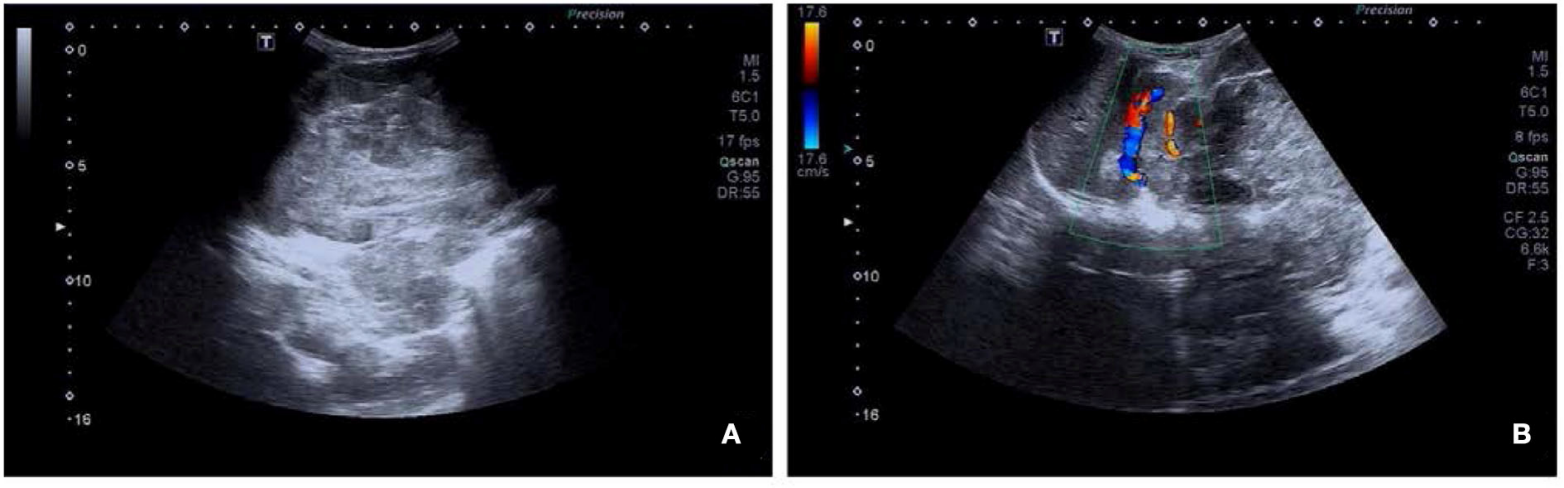
Ultrasound images of retroperitoneal alveolar rhabdomyosarcoma (ARMS). **(A)** A heterogeneous hypoechoic area of about 13.8 × 10.4 cm in the left upper abdomen, with an unclear boundary with the left kidney. **(B)** Blood flow signal observed using color doppler flow imaging (CDFI).

A 3-year-old female was accidentally found with a large left upper abdominal mass for a day. By physical examination, a smooth, hard, painless, and firm mass of about 14 cm in the left upper abdomen was found. Laboratory test showed increased fibrinogen of 4.08 g/L (reference range 2–4 g/L), neuron-specific enolase (NSE) of 79.90 ng/mL (reference range 15.6–17 ng/mL), and tumor abnormal protein (TAP) of 135.60 (reference range 1–121). An ultrasound showed a solid mass of about 13.8 × 10.4 cm in the left upper abdomen, with an unclear boundary with the left kidney. Blood flow signal could be observed using the color doppler flow imaging (CDFI) technique (Figure 1).

**Figure 2:**
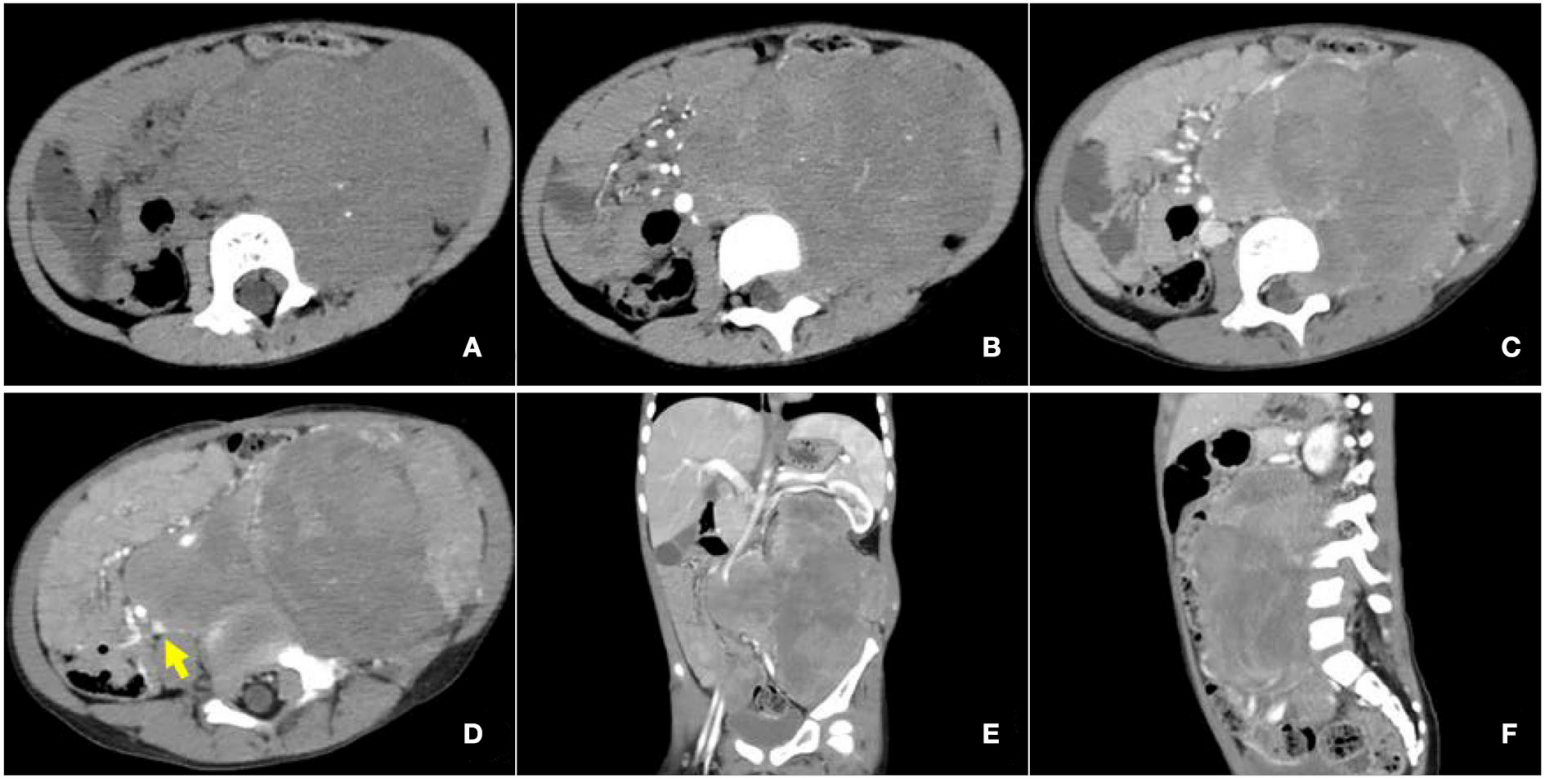
Enhanced computed tomography (CT) images of retroperitoneal ARMS. **(A)** A plain image with a CT attenuation value of about 47 HU; mottling calcification existing within the mass; **(B)** Arterial phase image with a CT attenuation value of about 50 HU; the mass supplied by branches of superior and inferior mesenteric arteries and areas of cystic degeneration and necrosis without enhancement existing within the mass; **(C–F)** venous phase image with a CT attenuation value of about 65 HU. Left renal vein reflux could be observed in the mass. The mass invaded the lumbar intervertebral foramen and compressed the left kidney, left abdominal bowel, and the inferior vena cava, and the abdominal aorta was also compressed and displaced to the right. The arrow illustrates the involvement of the inferior vena cava.

**Figure 3:**
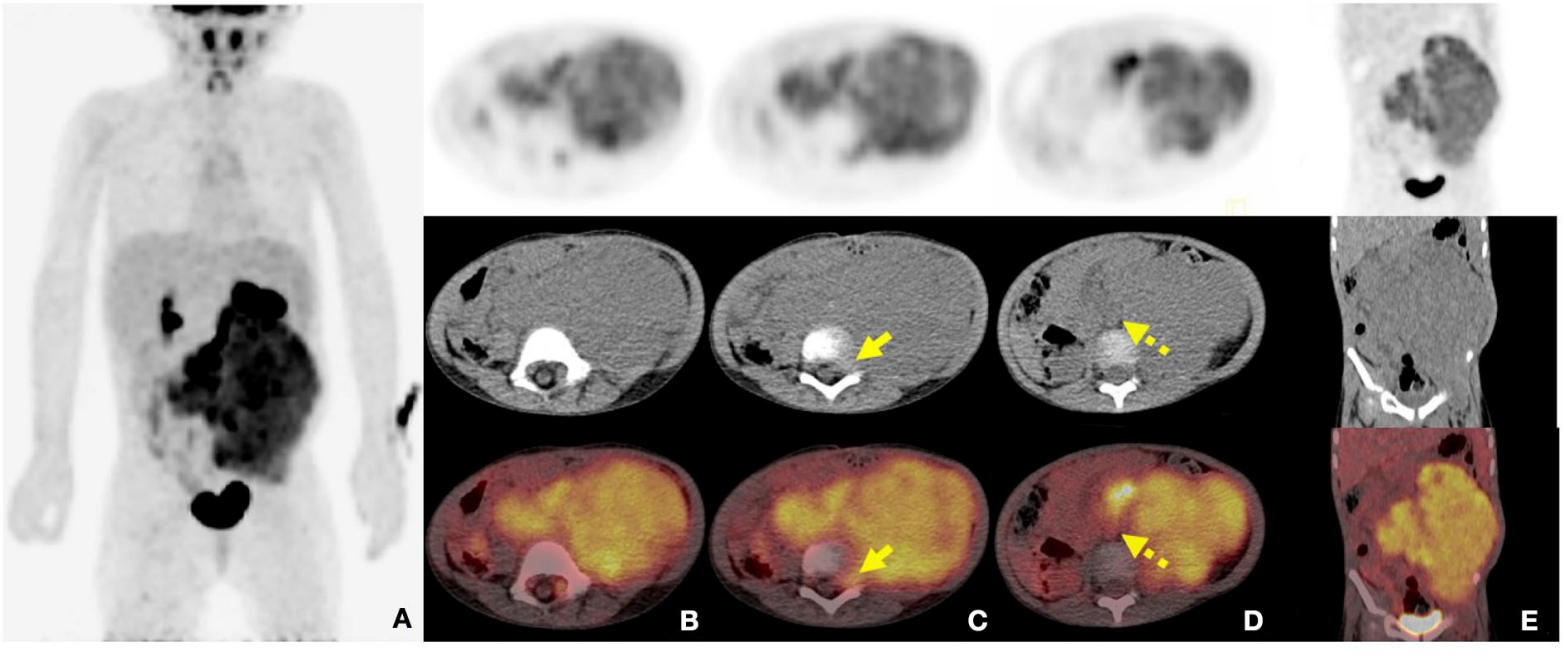
2-Deoxy-2-[fluorine-18]-fluoro-D-glucose (^18^F-FDG) positron emission tomography combined with computed tomography (PET/CT) images of retroperitoneal ARMS. **(A)** The whole-body maximum density projection showing hypermetabolic areas in the left retroperitoneum; **(B)** The axial images showing the concentrated distribution of radioactivity in soft tissue mass, with maximum standardized uptake value (SUV_max_) of about 7.0, the maximum layer of 8.8 × 10.3 cm, and a CT value of about 39 HU; **(C)** The axial images showing invasion of the left L3-5 intervertebral foramen and protrusion into the spinal canal, with unclear boundary with the spinal cord (short arrows); **(D)** The axial images showing the involved retroperitoneal lymph nodes (dash arrows); **(E)** The coronal image showing the lesion extending from the level T12 to the left acetabulum.

Contrast-enhanced CT examination indicated a huge soft tissue mass, measuring 12.3 × 7.4 cm at the larger section, in the left retroperitoneum extending superiorly to the level of the left hilus renalis and inferiorly to the left acetabulum in the pelvic cavity, with intrusion into the lumbar foramens, compression and squeezing of the left kidney and left abdominal bowel, local compression and stenosis of the inferior vena cava, and the abdominal aorta was displaced to the right side by compression (Figure 2). The tissue mass showed mottling calcification, cystic degeneration, and necrosis. The patient was injected with 0.1 mCi/kg of ^18^F-FDG after 6 h of fasting, and PET/CT images were acquired 60 min later. The tissue mass with a high FDG uptake was found using ^18^F-FDG PET/CT in the left retroperitoneum from the level of T12 to the left acetabulum, with the maximum standardized uptake value (SUV_max_) of about 7.0 and a CT value of about 39 HU. The tumor invaded the left L3-5 intervertebral foramina and protruded into the spinal canal, with an unclear boundary with the spinal cord (Figure 3). The abdominal cavity and retroperitoneum showed enlarged lymph nodes, with a SUV_max_ of about 6.4 and the largest size of about 2.0 × 3.0 cm. No obvious abnormal FDG uptake was observed in the lung and bone.

**Figure 4:**
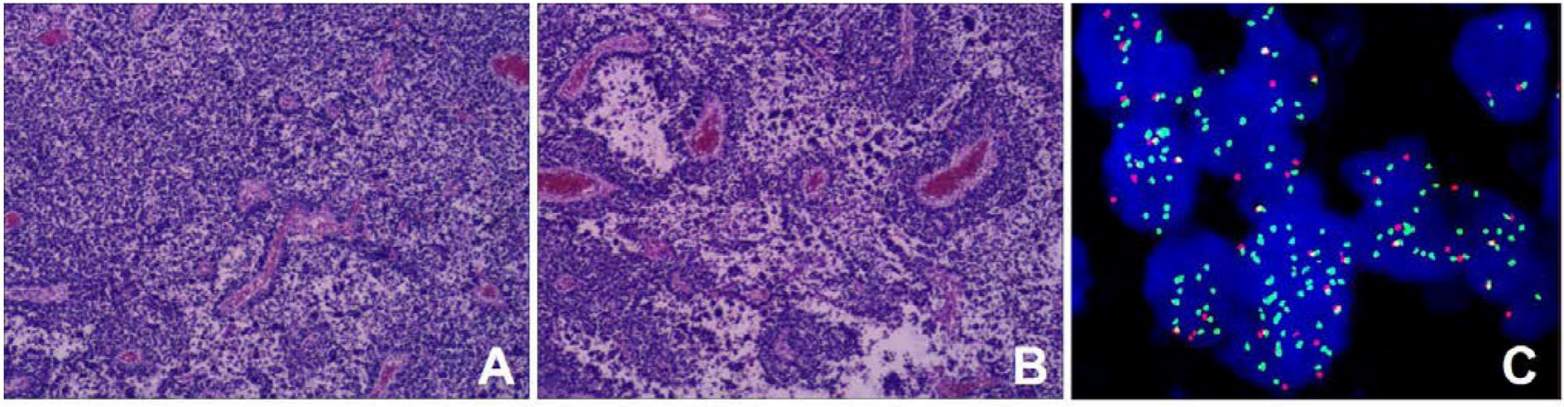
Histopathological results. **(A)** Tumor cells were round or oval with hyperchromatic nuclei and nuclear fission (HE staining); **(B)** Cells forming patterns resembling pulmonary alveoli, and fibrous vascular tissue between the alveoli (HE stain) [magnification (A-B) ×100]; **(C)** The fork head in rhabdomyosarcoma (FKHR) gene rupture was positive (fluorescence *in situ* hybridization) [magnification ×1000].

Retroperitoneal tumor resection and vascular repairing operation were performed 10 days after admission. The tumor was found to extend superiorly to the left hilus renalis, rightwards to cross the spine, and inferiorly to the level of the internal inguinal ring. It surrounded the abdominal aorta, the left iliac vessel, and the inferior vena cava, while locally infiltrating and growing in the inferior vena cava. After vessel clamping, the inferior vena cava segment invaded by the tumor was resected. Since the patient's family refused artificial vascular replacement, the inferior vena cava, at the level of the left and right iliac artery branches, and the left and right iliac veins were ligated. Histological results showed small round malignant cell with characteristic alveolar structures (Figures 4A,B). Immunohistochemical staining revealed myogenin (+), LCA (–), EMA (–), AE1/AE3 (–), Syn + (partly), NSE (–), CD99 (–), Fli-1 (–), Ki-67 (+60%), PAX-8 (+), CD56 (+), CgA (foci+), Desmin (+), Myo D1 (+), WT-1 (+), PD-1, S-100 (–), and PAX-5 (+). Fluorescence *in situ* hybridization revealed fork head in rhabdomyosarcoma (FKHR) rupture (Figure 4C). The final diagnosis was confirmed as ARMS with TNM stage 3 and IRS stage III, and the level of risk was high. Then, the patient received chemotherapy comprising vincristine, dactinomycin, and cyclophosphamide (VAC therapy) with supportive treatments to alleviate adverse reactions to chemotherapy. A combination of vincristine and irinotecan (VI) was administered 1 month later, and VAC was repeated 2 months later. The patient has survived for 9 months since the initial diagnosis with no recurrence and obvious complications.

## Discussion

Rhabdomyosarcoma is the most common extracranial solid tumor in the pediatric population after neuroblastoma and Wilms tumor (8). A total of 63% of RMS occur in children under 10 years old, and the incidence rate reaches the peak between 2 and 5 years old (11). ARMS is a relatively rare subtype with a poor prognosis, of which the estimated 10-year overall survival (OS) rate is 29.4% compared with 52.1% in the nonalveolar type (12). The retroperitoneum is not the typical primary site of RMS, with ~8% of the incidence of all sites (13). Approximately, 20% of the patients present with distant metastatic disease at diagnosis, and lung, bone, and bone marrow are the commonly involved sites (6, 14).

We searched the literature in the PubMed database from 1997 to 2022 using the keywords containing alveolar rhabdomyosarcoma and retroperitoneal. In total, 5 available case reports were found. We summarize the case reports in Table 1 (7, 8, 15–17). Of these cases, male children seem to be the more vulnerable group, which is consistent with overall incidence (male:female ratio of 1.51, 95% confidence interval (CI): 1.27–1.80) (18). Clinical symptoms are variable, depending on the size and location of the retroperitoneal mass and the presence or absence of distant metastases. The tumor squeezing certain organs can cause relevant performance, and the huge mass often invades vital organs such as the kidneys, aorta, inferior vena cava, and bilateral iliac vessels. However, patients can sometimes present with a mass and no notable symptom similar to the case we described here, causing a delay in diagnosis. It should be noticed that retroperitoneal ARMS appeared to have the potential to infiltrate the vertebrae and extend into the spinal canal. This could provide opportunities for tumors to invade the intervertebral foramen and spread to the spinal cord and even the brain as reported in the case study 2 (8). Thus, it argues for more attention on the careful and precise detection of the tumor intruding into the spinal canal and metastatic to the central nervous system.

**Table 1:** Characteristics of patients with retroperitoneal alveolar rhabdomyosarcoma (ARMS) in the literature review.

**Case**	**Authors**	**Patient sex**	**Age**	**Primary sites**	**Clinical symptom**	**Local invasion**	**Distant metastatic**	**Image methods**	**Management**	**Prognosis**
1	Ito et al. (7)	M	11 years	Retroperitoneum	Back pain	Adventitia of the aortic wall	None	MRI	Surgery + chemotherapy + radiotherapy	Alive at 12 month
2	Kline et al. (8)	M	14 months	Right retroperitoneum	Mass and irritability	Spinal canal	Bone marrow, lung and leptomeningeal metastatic	CT, MRI	Surgery + chemotherapy	Died after 2 month
3	Mariko Kinoshita et al. (17)	M	3 years	Retroperitoneum	Abdominal distention and constipation	Pleural space	Liver and multiple lymph nodes	^18^F-FDG PET/CT	Chemotherapy + radiotherapy	Alive at 22 month
4	Lugen Chen et al. (15)	M	13 years	Right retroperitoneum	Skin rash, petechiae, and ecchymoses over lower extremities and abdominal pain	–	Lymph nodes and bone marrow metastatic	CT	Chemotherapy	Alive at 8 month
5	Michiyuki Hakozaki et al. (16)	M	10 years	Left reniportal-retroperitoneal, groin and left gluteus maximus	Pain in the left buttock	–	Diffuse bone metastatic	CT	Surgery + chemotherapy + radiotherapy + peripheral blood stem cell transplantation	Died after 21 month

MRI, magnetic resonance imaging; —, No details in the report.

Alveolar rhabdomyosarcoma recurrently, of ~80%, harbors chromosomal translocations including a *t*(2;13)(q35;q14) or a *t*(1;13)(p36;q14), which can generate fusion genes PAX3- and PAX7-FOXO1 respectively. And proteins produced by these fusion genes can function as oncoproteins promoting the proliferation and apoptosis of tumor cells (6, 19). The diagnosis of ARMS requires histology and molecular pathology studies of the tumor tissue (6). ARMS is typically composed of densely packed, small, and round cells aggregating in areas at the edges of fibrous septa forming structures such as pulmonary alveoli (1, 20). Immunohistochemical markers include myogenic markers such as MyoD (myf3) or myogenin (myf4), myosin, myoglobin, muscle-specific actin, or desmin (21).

Medical imaging provides noninvasive methods which are essential for the evaluation of patients with ARMS. The sonographic feature of ARMS is substantive hypoechoic or complex-echoic mass. CDFI shows rich and disorderly color blood flow signals within the mass. It often appears as an equal or a slightly low-density mass in plain CT, with unclear borders. The tumors usually grow rapidly, and necrosis, as well as cystic degeneration, can be seen in the lesions as a result of the insufficient blood supply. Enhanced CT scan shows heterogeneous enhancement and sometimes rim-like enhancement. Areas without enhancement are tissues with necrosis and cystic degeneration. Hemorrhage and calcification occur rarely, but mottling calcification was observed in this case. Moreover, CT can detect adjacent bone involvement but ARMS frequently destroy the bone. PET/CT reveals increased glucose metabolism of ARMS. As an advanced technology, PET/CT could provide more information about the lesions than conventional imaging detection methods. ^18^F-FDG PET/CT imaging is useful for initial assessment, monitoring treatment response, and detection of recurrences with better accuracy for identifying primary sites, lymphatic involvement, and distant metastases (22, 23). Local lymph node metastasis has been considered a strong prognostic factor, calling for an emphasis on desirable detection modalities of lymphatic involvement (24). Compared with conventional imaging techniques, such as ultrasound, CT, and magnetic resonance imaging (MRI), PET/CT performs better in detecting lymph nodal metastasis with higher sensitivity and specificity (25). ^18^F-FDG PET/CT can estimate the function and nature of nodes through the level of glucose metabolism in tissues and can help with accurate localization of the involved lymph nodes. In a prospective study by Völker et al. (25), the detection of involved lymph nodes using ^18^F-FDG PET/CT reached a sensitivity of 93%, whereas conventional imaging modalities were only 36%. Ricard et al. (26) reported that ^18^F-FDG PET/CT found 19 involved lymph nodes in 4 patients vs. 12 nodes by MRI and CT, and therefore, the results of PET/CT led to alteration of the lymph node staging and treatment strategies in some patients. Our case also observed similar advantages of PET/CT for discovering retroperitoneal lymphatic metastases, whereas negative in ultrasound and CT tests. The more accurate staging of regional lymph node involvement will benefit risk stratification and treatment decisions in patients with RMS. PET/CT also shows some potential superiorities in finding tumor invasion into the spinal canal. When evaluating the spinal canal involvement, all background tissues, including paraspinal musculature, vertebrae, spinal cord, nerve roots, and CSF, demonstrate relatively low metabolic activity using ^18^F-FDG PET/CT, thus making it possible for differentiation between normal tissues and lesions (27, 28). PET/CT allows for the identification of soft-tissue involvement such as neural foramen invasion and epidural extension of tumor in malignant involvement of the spine (29). However, PET/CT is inferior to MRI when used to detect spinal cord involvement. In recent years, the integrated PET and MR (PET/MR) imaging modality has been rapidly developed with the combined superiorities of quantification of radioactive tracer metabolism provided by PET and outstanding soft tissue contrast by MR (30). The value of PET/MR in clinical applications remains to be established, and we hope this innovative technology will provide more accurate diagnosis and ultimately improve patient prognosis. In addition, a study illustrated that metabolic parameters obtained from baseline PET/CT were potential to select patients sensitive to treatment (31). Features of patients including unfavorable sites of the primary tumor, older patient age at initial presentation, the alveolar subtype, and regional lymph node involvement are considered to be poor prognostic factors for RMS (5, 32). Unfavorable sites include the prostate and bladder, cranial parameningeal sites, extremities, trunk, retroperitoneum, and other sites (13). Moreover, ^18^F-FDG PET/CT may be an added prognostic predictor in RMS. High SUV_max_ value is more prevalent among patients with less favorable features including unfavorable primary sites, alveolar pathology, and high-risk group (33). A study found that during diagnosis, patients with SUV_max_ of <9 had an improved 3-year progression-free survival (62% of patients with SUV_max_ of <9 vs. 39% of patients with SUV_max_ of ≥9, *p* = 0.02) (34). In our case study, the SUV_max_ of 7.0 might be associated with the patient's favorable prognosis.

Retroperitoneal ARMS should be differentiated from neuroblastoma. Neuroblastoma is the most common extracranial solid tumor in the pediatric population and almost 70% of the patients have abdominal neuroblastoma (35, 36). Furthermore, neuroblastoma may arise from paraganglia and is likely to protrude into the spinal canal through the neural foramina. In addition to symptoms caused by the abdominal mass such as abdominal pain and fullness, patients usually present with elevated levels of vanillylmandelic acid (VMA) and homovanillic acid (HVA). The characteristic PET/CT findings of neuroblastoma in children include large size, mixed density with calcification, necrosis, and cystic degeneration, which specifically show peripheral hypermetabolic areas and central hypometabolic areas in the tumor, indicating central necrotic and cystic lesions. PET/CT is superior at revealing lymph nodes and distant organ metastases, which can provide an objective imaging basis for preoperative diagnosis and accurate staging of neuroblastoma in children, over conventional imaging. Calcification could be seen in almost 90% of cases, appearing as sandy, spotted, and mass shapes, which is a characteristic manifestation of neuroblastoma (37). ^123^I-Metaiodobenzylguanidine (MIBG) plays an important role in the diagnosis of neuroblastoma with an accumulation of MIBG in the lesions (38). However, the limited sensitivity of ^123^I-MIBG needs to be improved. A prospective study by Piccardo et al. demonstrated that ^18^F-3,4-dihydroxyphenylalanine (^18^F-DOPA) PET/CT is more sensitive in detecting primary tumors, soft tissue metastases, and bone and bone marrow metastases than ^123^I-MIBG (39). The diagnosis of neuroblastoma should be confirmed by biopsy. In this case, the diagnosis of ARMS was not definite until the results of postoperative histology distinguished it from neuroblastoma.

Overall, the cure rate of RMS could be increased with the improvements in risk stratifications and multimodal treatment including surgery, chemotherapy, and radiotherapy (40, 41). As for retroperitoneal RMS, surgery, especially radical resection, is the principal choice, with a longer median OS than palliative surgery and conservative treatment (18 vs. 6 months) (9).

## Conclusion

Retroperitoneal ARMS is relatively rare and characterized by its unfavorable outcome with the potential to involve vital organs and intrude into the spinal canal, and even spread to CNS. It should be noted that the retroperitoneal mass may be misdiagnosed as neuroblastoma and a biopsy is necessary for the final diagnosis. Accurate staging using PET/CT would contribute to better risk stratifications and appropriate treatment individually.

## Data availability statement

The original contributions presented in the study are included in the article/supplementary material, further inquiries can be directed to the corresponding author.

## Ethics statement

Ethical review and approval was not required for the study on human participants in accordance with the local legislation and institutional requirements. Written informed consent from the patients/participants legal guardian/next of kin was not required to participate in this study in accordance with the national legislation and the institutional requirements. The study was approved by the Institutional Review Board at the First Affiliated Hospital of Zhengzhou University and Peking University First Hospital.

## Author contributions

YZ manuscript drafting. WH acquisition and analysis of the work and imaging data collection and analysis. LL imaging data collection and analysis and resources collection. YQ and HJ manuscript editing. ZC, QY, and LS formal analysis and resource collection. LK supervision and writing-review and editing. All authors met the requirements for authorship for the submitted version and agreed to its submission.

## Funding

This study was funded by the Beijing Science Foundation for Distinguished Young Scholars (JQ21025) and the Peking University Medicine Fund of Fostering Young Scholars' Scientific and Technological Innovation (BMU2022PY006).

## Conflict of interest

The authors declare that the research was conducted in the absence of any commercial or financial relationships that could be construed as a potential conflict of interest. Written informed consent was obtained from the minor's legal guardian for the publication of any potentially identifiable images or data included in this article.

## Publisher's note

All claims expressed in this article are solely those of the authors and do not necessarily represent those of their affiliated organizations, or those of the publisher, the editors and the reviewers. Any product that may be evaluated in this article, or claim that may be made by its manufacturer, is not guaranteed or endorsed by the publisher.
